# HPV self-sampling acceptability among women in Italy: preliminary results of a cross-sectional study

**DOI:** 10.1093/eurpub/ckac131.564

**Published:** 2022-10-25

**Authors:** S Angelillo, F Licata, EA Errico, R Maruca, S Freccia, R Pujia, G Di Gennaro, A Bianco

**Affiliations:** 1 Department of Health Sciences, University of Catanzaro, Catanzaro, Italy; 2 Department of Medical and Surgical Sciences, University of Catanzaro, Catanzaro, Italy

## Abstract

**Background:**

Secondary prevention measures have strongly contributed to the reduction of incidence and mortality of cervical cancer (CC) identifying women at high risk of developing it. This cross-sectional study aimed to investigate the acceptability of a home-based self-sampling methodology for Human Papilloma Virus (HPV) testing and the factors that may influence women’s preference.

**Methods:**

A random sample of women over the age of 50 years has been selected in Southern part of Italy. Data was collected through an anonymous self-administered questionnaire and included socio-demographic characteristics, knowledge of HPV infection and prevention measures, and attitudes towards the acceptability of self-collected cervico-vaginal sampling (CVS) and urine sampling (US).

**Results:**

Among the 321 women who completed the survey, more than two-thirds (73.7%) knew that CC is caused by HPV, only 68.9% knew that the HPV screening is useful for an early detection and diagnosis of CC, and 17% never had Pap-test or HPV-DNA test. Of the respondents, 67.9% declared that they preferred self-collected US for future HPV testing compared with clinician-taken cervical samples (CCS). The most common reasons reported for preferring US included that it was easier (54.8%), more convenient (28.7%), and less embarrassing (21.7%). Among those women who showed negative attitude towards self-collected US, 8 out of 10 (77.3%) expressed scepticism about its diagnostic performance. Only 37% of the sample preferred CVS, and this attitude is mainly attributable to the fear of not carrying out a correct self-sampling (71.2%) and to its underrated diagnostic performance (33.7%).

**Conclusions:**

The preliminary results suggest that US is more acceptable than CCS and CVS in Italy. Urinary HPV test presents similar accuracy of the latter tests to detect CC and its supply in the context of population-based screening programmes could improve adherence, reducing the cost and burden on physicians.

**Key messages:**

• Urine self-sampling could represent an innovative early detection approach to increase adherence to cervical cancer screening programmes.

• Further research is needed to assess whether the screening status and the strategy of self-samplers distribution could act as predictors of screening uptake.

